# The modular nature of bradykinin-potentiating peptides isolated from snake venoms

**DOI:** 10.1186/s40409-017-0134-7

**Published:** 2017-10-26

**Authors:** Juliana Mozer Sciani, Daniel Carvalho Pimenta

**Affiliations:** Laboratory of Biochemistry and Biophysics, Butantan Institute, Av. Vital Brasil, 1500, São Paulo, SP CEP 05503-900 Brazil

**Keywords:** *Bothrops jararaca*, Venom, Snake venom, Bradykinin-potentiating peptides, BPP, Modules

## Abstract

Bradykinin-potentiating peptides (BPPs) are molecules discovered by Sergio Ferreira – who found them in the venom of *Bothrops jararaca* in the 1960s – that *literally* potentiate the action of bradykinin in vivo by, allegedly, inhibiting the angiotensin-converting enzymes. After administration, the global physiological effect of BPP is the decrease of the blood pressure. Due to this interesting effect, one of these peptides was used by David Cushman and Miguel Ondetti to develop a hypotensive drug, the widely known captopril, vastly employed on hypertension treatment. From that time on, many studies on BPPs have been conducted, basically describing new peptides and assaying their pharmacological effects, mostly in comparison to captopryl. After compiling most of these data, we are proposing that snake BPPs are ‘modular’ peptidic molecules, in which the combination of given amino acid ‘blocks’ results in the different existing peptides (BPPs), commonly found in snake venom. We have observed that there would be *mandatory* modules (present in all snake BPPs), such as the N-terminal pyroglutamic acid and C-terminal QIPP, and *optional* modules (amino acid blocks present in some of them), such as AP or WAQ. Scattered between these modules, there might be other amino acids that would ‘complete’ the peptide, without disrupting the signature of the classical BPP. This modular arrangement would represent an important evolutionary advantage in terms of biological diversity that might have its origins either at the genomic or at the post-translational modification levels. Regardless of the modules’ origin, the increase in the diversity of peptides has definitely been essential for snakes’ success on nature.

## Background

Snakes are elongated, legless, carnivorous reptiles of the order Squamata and suborder Serpentes (Ophidia) found on every continent except Antarctica [1]. They are thought to have evolved from either burrowing or aquatic lizards, perhaps during the Jurassic period, with the earliest known fossils dating to between 143 and 167 million years ago. The diversity of modern snakes appeared during the Paleocene period (66 to 56 million years ago). The oldest preserved descriptions of snakes can be found in the Brooklyn Papyrus (450 BC), a manuscript from the ancient Egypt that systematically describes snakes and different treatments for snakebites [2].

Currently, there are circa 3400 snakes species, divided into 18 families. Although most species are non-venomous, those that have venom use it primarily to kill and subdue prey rather than for self-defense [3]. *Elapidae* and *Viperidea* are two examples of such families that might be dangerous to humans, once the specimens contain highly toxic venom [1].

Snake venom, which is a highly modified saliva produced by modified salivary glands, is injected by specialized hollow teeth. It is thought to have evolved from simple sets of proteins through gene duplication and natural selection, followed by functional diversification [4]. *Elapidae* venoms are, in general, more neurotoxic whereas *Viperidae* venoms act more on coagulation factors [5, 6].

*Bothrops* (Serpentes: Viperidae: Crotalinae) is an endemic genus to Central and South America, and contains 32 species. Among its members, *Bothrops jararaca –* native to Brazil, Paraguay and Northern Argentina – deserves credit for being the species from which Sergio Ferreira isolated the bradykinin-potentiating factors (BPF) in the 1960s [7]. Later, these factors were identified to be peptides, named bradykinin-potentiating peptides (BPPs), and were the molecular basis for the design of captopril, the world famous antihypertensive drug, developed by Cushman and Ondetti [8].

BPPs are proline-rich peptides, known to inhibit the angiotensin-converting enzyme (ACE). This enzyme is responsible for the conversion of angiotensin I into angiotensin II, which is a potent vasoconstrictor and hypertensive agent. By inhibiting ACE, BPPs inhibit angiotensin II formation, reducing the blood pressure [9]. Moreover, since this enzyme is able to cleave bradykinin as well (a hypotensive peptide), its inhibition is comprised of two different mechanistic targets (angiotensin and bradykinin) causing global hypotensive effects [10].

The first BPP was discovered by Ferreira and Rocha e Silva [11] and termed as such due to its pharmacological effect, i.e., literally potentiate/enhance the effect of bradykinin (BK), in comparison to the same BK dose administered prior to the BPP treatment, without presenting any hypotensive effect per se. Since that time, biological driven assays have led to the discovery of several BPPs from many sources, such as scorpion venoms, tree-frog skin secretions and in the venom of snakes from other species [12–16]. Nevertheless, since the term BPP actually describes an *effect* and not a structural motif, there are many related and unrelated peptide sequences that can elicit such BK potentiation (Table 1 presents some of these peptides). Therefore, this paper will focus solely on *snake BPPs.*

**Table 1:** Peptides displaying BPP *activity* isolated from different biological sources

Name (Source)	Sequence
BPP 11a (*Bothrops jararaca*)^a^	<EWPRPTPQIPP
^108-113^casein^b^ (*Bos taurus*)	EMPFPK
Phypo Xa (*Phyllomedusa hipochondrialis*)^c^	<EFRPSYQIPP
BPP-Si (*Scaptocosa raptoria*)^d^	<EAPWPDTISPP
K12j (*Buthus occitanus*)^e^	LRDYANRVINGGPVEAAGPPA
TsHpt-Ik (*Tityus serrulatus*)^f^	AEIDFSGIPEDIIKQIKETNAKPPA
LVV-Hemorphin-7^g^ (*Canis lupus familiaris*)	LVVYPWTQRF

<E: pyroglutamic acid

^a^ [17] ^b^ [31], ^c^ [30], ^d^ [32], ^e^ [29], ^f^ [12], ^g^ [33]

### Structure of BPPs

Snake BPPs are (mostly) < 14-mer proline-rich peptides (PRPs), presenting three distinct features: i) an almost invariable pyroglutamic acid at the N-terminus; ii) the presence of a Pro residue at the C-terminus for peptides containing up to five residues; iii) the presence of the tripeptide Ile-Pro-Pro at the C-terminus of >5-mer peptides containing up to 14 residues [17]. Therefore, the general structure pattern of BPPs would be:

$$ {\displaystyle \begin{array}{c}\hfill <E-{X}_n-P-{X}_n-P-{X}_n-I-P-P\hfill \\ {}\hfill \left(<E: pyroglutamic acid;X: any\  amino acid, but\  Cys\right)\hfill \end{array}} $$

With a rather limited amino acid substitution capacity, in order to fit in this criterion, it is not surprising that many BPPs are very similar, and also that the exact same peptide sequences can be found in different species of snakes. For instance, larger BPPs (> 10 amino acids) tend to have the tripeptide IPP at the C-terminus, whereas the shorter sequences (5, 6 and 7 mers) preserve only the N-terminal part of the scaffold presented above. Moreover, some larger BPPs possess the same N-terminal amino acid sequence as others shorter BPPs. One example comprises BPPs 5b (<EWPRP) and 9a (<EWPRPQIPP), in which BPP 5b is, in fact, the N-terminus of BPP 9a [17]. BPP 5b is also very similar to BPP 5a (<EKWAP), the one that served as template for the development of captopril [11]. Naturally, these similarities and differences also occur at the pharmacological and enzymatic levels, yielding different *k* values according to the substituted amino acid and the performed biological assay [18].

### Modular sequences

In 2004, Ianzer et al. [17] identified several BPPs, either ‘new’ or ‘old’ peptides (previously described) in *B. jararaca* venom, using both biological monitored screening and the incipient (at the time) de novo peptide sequencing by tandem mass spectrometry (MS/MS), after a straight forward two-step liquid chromatography sample preparation procedure. Currently, when revisiting that paper [17], these authors could notice that two out of the five new sequences described were mere variations of already known peptides, with an extra -WAQ- sequence buried within.

This observation triggered the authors’ curiosity to revisit other studies as well, particularly the ones containing description of new/novel BPPs. The work by Pimenta et al. [19] analyzed several *B. jararaca* venom samples, from both siblings born and raised in captivity and wildlife captured individuals. It describes that the peptide contents of the venom is different in male and female individuals. Authors sequenced the female-exclusive peptides and observed that those were the known BPPs 9a, 10b, 10c and 11a, but lacking the C-terminal peptide sequence -QIPP. They concluded – at the time – that those were ‘cleaved/processed’ peptides present only in female individuals. However, in light of this new proposed point of view, it would be more likely that the -QIPP ‘module’ would be added in males, rather than removed in females. A few points corroborate this idea: i) the ‘processed’ QIPP peptide was not observed in any venom of any individual, by any technique (ESI and/or MALDI) in that study; and ii) the processing site would involve an enzyme capable of cleaving the Pro-Gln peptide bond, which is quite specific. A query for this specificity at the MEROPS data base [20] yields few enzymes, including papain and other cysteine peptidases, as well as prolyl oligopeptidase (POP) and some matrix metallopeptidases (MMPs), including a disintegrin and metallopeptidase (ADAMs). Taking into account that no POP or cysteine peptidase were ever found in snake venom and that there are published reports demonstrating that the MMPs/SVMPs (snake venom metallopeptidase) present in the venom are catalytically inactive due to a three-component inhibition system (low pH, Ca^2+^ chelation and peptide inhibition; [21]), the -QIPP module *addition* in males turns to be much more feasible than the -QIPP *removal* in females, according to our current proposal.

Further exploration of published data on snake BPPs led us to identify another ‘module’: -AP-. This dipeptide module, however, possess an interesting characteristic: it is mainly located at the C-terminal of the BPPs, as one can observe for BPP-AP from *B. jararacussu* (<EARPPHPPIPPAP) and BPP 12d from *B. cotiara* and *B. fonsecai* (<ENWPHPPMPPAP). Zelanis et al. [22], when analyzing the peptidome of *B. jararaca*, were able to identify the presence of BPP 11e with AP at the C-terminal in both adults and newborn specimens. This same study presents BPPs with extra amino acids, but we could not classify them as ‘modules’ (yet) and neither did the authors, which considered those peptides to be mere ‘variations’ and/or ‘redundancies’ of the snakes’ peptide repertoire. Based on this rationale, the very first/primordial modular BPP would be BPP 5a (<EKWAP), which is nothing more than the intrinsic metallopeptidase inhibitor <EKW [21] with the -AP- module addition.

Nevertheless, some *Crotalus* sp*.* BPPs present the -AP- module within the sequence, such as BPPs 11b and 11c (<EGGAPWNPIPP and <ESAPGNEAIPPA, respectively). BPP 13d seems to have duplicated this modular insertion: <EGRAPHPPIPPAP. Moreover, a very interesting new BPP isolated from *Bitis gabonica rhinoceros* (APQERGPPEIPP) is, so far, the sole BPP with the -AP- module at the N-terminal [22–25].

Other internal sequences that appear to be modules are: -PRP- and -PHP-. BPP 10b–F and 10c–F would fit in the same ‘primordial’ BPP category as BPP 5a. For 10b–F and 10c–F would be the MMP inhibitor <ENW, plus the -PRP- and -PHP- motifs. Nevertheless, these are the only examples of the existence of these modules (−PRP- and -PHP-), e.g., these peptides have been actually and independently identified. No other desPRP or desPHP BPPs have been described so far.

### The roadmap to the BPPs

Through analyses of the BPP sequences, based on sequence alignments and other structural features, we propose that there are two possible structural motifs for the BPPs: the ‘mandatory’ and the ‘optional’. The mandatory motifs would be:

$$ {\displaystyle \begin{array}{rrr}\hfill \left(N- terminal\right)& \hfill (Internal)& \hfill \left(C- terminal\right)\\ {}\hfill <E/< EXW& \hfill - PZP-& \hfill PPIPP\\ {}\hfill \left(X=N\  or\ K;Z=R,H\  or\ G\right)& \hfill & \hfill \end{array}} $$

whereas the optional motifs would be:

$$ AP- WAQ- QIPP $$

Therefore, by arranging and combining the modules above, one ought to be able to ‘recreate’ all described BPPs and, eventually, predict the existence of undiscovered molecules.

**Fig. 1:**
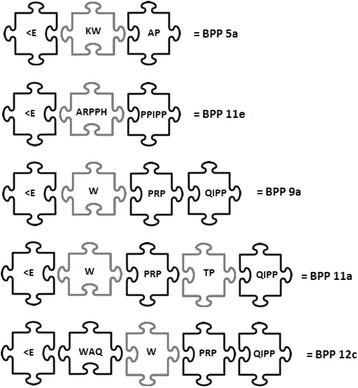
Actual modular BPPs. In black: mandatory/optional pieces. In grey: additional residues

Examples of actual peptides bearing this modular assembly are: (Fig. 1).

Therefore, other peptides, not limited to these, must exist, based on different/random combinations of mandatory and optional modules. Below, we propose a made up sequence comprised of one mandatory and two optional motifs:

$$ < EKW+ AP+ QIPP $$

When querying UniProt, there is close call for the *Echis ocellatus* SVMP inhibitor 02D01 (UniProt A8YPR6), which is a precursor of many peptides, including this sequence:

$$ {\dots}^{159}{QKWQPQIP}^{166}\dots $$

One must take into account that the pyroglutamic acid is a post-translational modification that is normally annotated as glutamine (Gln; Q) in the precursor protein in the deposited sequences.

In fact, A8YPR6 precursor seems to be very much like the *B. jararaca* BPP/C-type natriuretic peptide precursor (UniProt Q6LEM5), i.e., a multiple peptide precursor, containing several BPPs. The ‘test’ performed with the sequence above aligns with ten more internal peptides from this *E. ocellatus* precursor (39–48; 51–60; 75–84; 87–96; 99–108; 111–120; 135–144; 147–156; 63–72; 244–246; Uniprot numbering; listed in decreasing score order).

Another test subject for our hypothesis could be:

$$ < EKW+ PRP+ AP+ QIPP $$

This yields good matching to BPP 11, from *B. neuwiedi* (UniProt P0C7S5).

$$ {\dots}^2{WPRPTPQIPP}^{11} $$

As well as other lower scores matches among several other BPPs and BPP/C-natriuretic peptide precursors, as commented above, there is little room for sequence variation under the rigid constrains of the canonical BPP. This exercise would go on and on and as our hypothesis seems to be valid.

### Module distribution

**Fig. 2:**
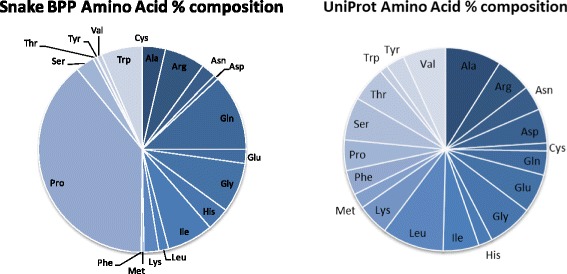
Amino acid percent composition of the samples

Based on the 87 currently available deposited snake BPPs (Table 2), we have performed some sequence feature analyses in order to evaluate whether our modular proposal is subsided by the actual amino acid composition/distribution. First, we have compared the percent amino acid distribution in the whole proteome (UniProt Release 2016_10 of 2 Nov. 2016 of UniProtKB/TrEMBL containing 70,656,157 sequence entries, comprising 23,670,752,099 amino acids; https://web.expasy.org/docs/relnotes/relstat.html) with the amino acid composition of the peptides listed in Table 2. Figure 2 presents the distribution of the percent composition of the samples.

**Table 2:** Snake BPPs analyzed

**BPP10a**	QSWPGPNIPP	**BPP-11b-CROA**	QGGWPRNPIPP
**BPP6a**	QSWPGP	**BPP-11c-CROV**	QSAPGNEAIPP
**BPP13a + QQWA**	QQWAQGGWPRPGPEIPP	**BPP-11-BOTAL**	QWPDPSSDIPP
**BPP13a + QWA**	QWAQGGWPRPGPEIPP	**BPP-11**	QGGAGWPPIPP
**BPP13a**	QGGWPRPGPEIPP	**BPP-11-NEW**	QWPRPTPQIPP
**BPP10c + QQWA**	QQWAQNWPHPQIPP	**BPP-13a**	QGGWPRPGPEIPP
**BPP10c**	QNWPHPQIPP	**BPP-2-Glo**	QGRPPRPHIPP
**BPP10c-F**	QNWPHP	**BPP-POL-236**	QLWPRPQIPP
**BPP11b**	QGRAPGPPIPP	**BPP-5-NEW**	EEGGSPPPVVI
**BPPIIb**	QGRAPHPPIPP	**BPP-7b**	QNWPSPK
**BPP5a**	QKWAP	**BPP-7c**	QRWPSPK
**BPP12b**	QWGRPPGPPIPP	**BPP-8a**	QNAHPSPK
**BPP-APL**	QARPPHPPIPPAPL	**BPP-9a**	QWPRPQIPP
**BPP11e**	QARPPHPPIPP	**BPP-10d**	QNWPHPPMPP
**BPP11eAP**	QARPPHPPIPPAP	**BPP-10e**	QNWPSPKVPP
**BPP-AP**	QARPPHPPIPPAP	**BPP-10f**	QRWPSPKVPP
**BPP-A-AK**	QGRPPGPPIP	**BPP-11d**	QGRPPGPPIPP
**BPP-C-AK**	QGLPPGPPIPP	**BPP-11f**	QNAHPSPKVPP
**BPP-B-AK**	QGLPPRPKIPP	**BPP-11 g**	QARPRHPKIPP
**BPP-Ahb1**	QKWDP	**BPP-11 h**	QGRHPPIPPAP
**BPP-Ahb2**	QPHESP	**BPP-11i**	QNGPRPIGIPP
**BPP-1LM**	WPPRPQIPP	**BPP-11j**	QNRHPPIPPAP
**BPP-2LM**	QKPWPPGHHIPP	**BPP-Pb**	QTLLQELPIPP
**BPP-3LM**	QEWPPGHHIPP	**BPP-12a**	QGWAWPRPQIPP
**BPP-4LM**	QKKWPPGHHIPP	**BPP-12b**	QLGPPPRPQIPP
**BPP-5LM**	QKWDPPPISPP	**BPP-12d**	QNWPHPPMPPAP
**BPP-12c**	QWAQWPRPQIPP	**BPP-12e**	QARPRPGPKIPP
**BPP-14a**	QWAQWPRPTPQIPP	**BPP-13a**	QGGWPRPGPEIPP
**BPP-Tf1**	QSKPGRSPPISP	**BPP-13b**	QGGLPRPGPEIPP
**BPP-Tf2**	QWMPEGRPPHPIPP	**BPP-13c**	QGRPPHPPIPPAP
**BPP-Tf3**	QGRPRSEVPP	**BPP-13d**	QGRAPHPPIPPAP
**BPP-2-Sisca**	QNWKSP	**BPP-TmF**	QGRPLGPPIPP
**BPP-Cdt1a**	QWSQRWPHLEIPP	**BPP-Phypo-Xa**	QFRPSYQIPP
**BPP-Cdt1b**	QRWPHLEIPP	**BPP-VIPAS**	QGWPGPKVPP
**BPP-Cdt2**	QNWKSP	**BPP-5b-9a-F**	QWPRP
**BPP-Cdt3**	QARESP	**BPP-10b-F**	QNWPRP
**BPP-1CRO**	QRWPHLEIPP	**BPP-11a-F**	QWPRPTP
**BPP-2CRO**	QNWKSP	**BPP-F-AK**	QLWPRPHIPP
**BPP-10b**	QNWPRPQIPP	**BPP-3-new**	QGGWPRPGPEIPP
**BPP-Tg1**	QEKPGRSPPISP	**BPP-S412**	QGGPPRPQIPP
**BPP-12a**	QQWPRDPAPIPP	**BPP-S51**	QWGQHPNIPP
**BPP-7a**	QDGPIPP	**BPP-11a**	QQWPPGHHIPP
**BPP-S**	QAPWPDTISPP	**BPP-11b-CRO**	QGGAPWNPIPP
**BPP-1-Glo**	QGRPPGPPIPP		

N-terminal Q denotes pyroglutamic acid

Most BPP’s nomenclature depends on: i) the number of amino acids of the peptide (BPP-5 has five amino acids, BPP-10, ten amino acids and so on); ii) the order of discovery of the peptide. BPP-10a was the first to be described, then BPP-10b, 10c, and so on; and iii) a possible extra feature. For instance, −F indicates peptides present only in females. -AP denotes an extra dipeptide AP added to an existing sequence

In bold, the peptide names as deposited in the UniProt database

One can clearly observe that the UniProt amino acid composition follows an almost identical distribution, being Leu the most abundant amino acid (~10%) and Cys and Trp the rarest ones (~1%), e.g., a ten-fold maximum variation. On the other hand, the snake BPPs amino acid composition is rather heterogeneous. Proline is the most copious amino acid, corresponding to circa 40% of the total. Not taking into account that – so far – no BPP has ever been described containing a Cys residue, Phe and Tyr are the least common amino acids, in a 40:1 relationship. Although Gln appears at the second position, this figure comprehends both glutamine and pyroglutamic acid, a consequence of the UniProt notation for the N-terminal substitution. The significantly abundant amino acids ranking second are Gly, Ile, Trp and Arg that together (255 AA) add up to more than the remaining 13 amino acids (193 AA). According to this analysis, the BPPs can – undoubtfully – be classified as proline-rich peptides.

Next, we have tested whether these amino acids are randomly distributed or would they be arranged in preferred groups. Table 3 presents the average dipeptide composition of all possible amino acids but Cys. The frequency is presented in percent values together with a gray bar corresponding to the figure for better visualization. One can observe that Pro not only is the most common amino acid but also that the dipeptide Pro-Pro is the most frequent amino acid combination, followed by Ile-Pro, Trp-Pro and Arg-Pro; not surprisingly the same second-most abundant amino acids. This table provides a clear depiction of the preferred amino acid pattern arrangement instead of a random distribution.

**Table 3:**
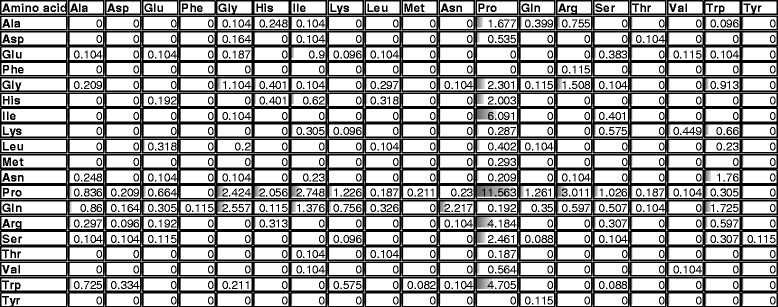
Dipeptide frequency distribution

**Fig. 3:**
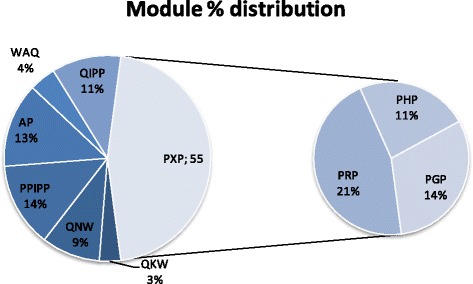
Percent distribution of the modules identified in snake BPPs

After performing such ‘quality control’ steps, it is our understanding that the amino acids present in snake BPPs do follow a pattern and that this pattern can be categorized into ‘modules’ that can also be analyzed according to their distribution, based on the current availability of peptide sequences. Figure 3 shows the percent distribution of the modules we were able to identify in the snake BPPs. Furthermore, this figure group the PRP, PHP and PGP modules in a ‘generic’ PXP module that corresponds to more than half of the modular instances detected. If one groups the IPP present in the QIPP and PPIPP modules, one reaches 25% (less than half of the PXP modules distribution). This observation becomes quite important when the majority of the papers dealing with snake BPPs state that ‘canonical BPPs are proline rich peptides presenting a pyroglutamic acid at the N-terminal and the IPP tripeptide at the C-terminal’. Although correct, this sentence is not accurate in terms of the representativeness of the actual motifs. Perhaps a better introduction to these peptides would be: ‘*canonical snake BPPs are proline-rich peptides presenting the PXP motif, a pyroglutamic acid at the N-terminal and the IPP tripeptide at the C-terminal*’.

## Conclusion

With no available knowledge on the molecular genetics of any *Bothrops* sp. snake (the main BPP producing snakes), nothing can be inferred about the chromosomic origins of these modules; nevertheless, their existence and participation in the ‘creation’ of the different sequenced BPPs is unequivocal.

On the other hand, by synthesizing peptides as modules, the variability increases several times, what would also increase the number of molecules in venom. The variability of molecules leads into improved biological activities, important for more efficient prey envenomation. This would represent a great evolutionary advantage, once the animal genome would not be as complex as the number of secreted peptides, similar to the de novo antibody production [26]. Nevertheless, complimentary genetic studies are needed to evaluate this hypothesis. What has been previously postulated is that the snake venom gland has undergone a process of gene recruitment and duplication, resulting in toxic/enzymatic variability [27].

Kininogens have already been reported in the snake plasma and different kinins isolated from fish, amphibians and birds [28]. Altogether, there might be possible that there were original kinins in the snake plasma (although none has been described yet), derived from the kininogen, which have undergone a recruitment process on the venom gland. In the gland, the kinins would have turned into the BPPs and kininogen, into the BPP/C-type natriuretic peptide precursor. This would explain why the long sought ‘endogenous BPP’ has never been found in mammals, no matter how hard some groups have tried [29].

In fact, according to our understanding and the currently proposed module approach, kinins would be the ‘original BPPs’. It seems that the Amphibia have already experimented this theme by long secreting bradykinin-related peptides (BRP) through the skin. We have previously explored some structural features of these BRPs when describing three new BRPs in *Phyllomedusa* [30] and concluded – once more – that there is very little room for sequence variation when there are so many structural constrains involved. The same happened when we described the first canonical BPP not from snake venom, but from the tree frog *P. hypochondrialis* (BPP Phypo-Xa) [13]. The structural features analyses of this 10-mer canonical BPP, known at that time, points out to a very high degree of conservation, compared to others BPPs. Unfortunately, few kininogens and BPP/C-natriuretic precursor sequences are currently available to prove this hypothesis by constructing phylogeny trees.

In sum, it is our understanding the snake BPPs follow the modular construction pattern and, as far as new sequences are discovered, more patterns may be perceived. This modular design would explain the variability of peptides present in the venom and the consequent evolutionary success of these animals.

## Abbreviations

ACE: Angiotensin-converting enzyme
ADAMs: A disintegrin and metallopeptidase
BK: Bradykinin
BPF: Bradykinin-potentiating factors
BPPs: Bradykinin-potentiating peptides
BRP: Bradykinin-related peptides
MMPs: Matrix metallopeptidases
MS/MS: Tandem mass spectrometry
POP: Prolyl oligopeptidase
PRPs: Proline-rich peptides
SVMPs: Snake venom metallopeptidase
